# Colloidal Suspensions Displaying Anomalous Phoretic Behavior: Field and Mobility Reversal

**DOI:** 10.1021/acs.langmuir.2c01316

**Published:** 2022-09-06

**Authors:** Vincenzo Tricoli, Fulvio F. Corinaldesi

**Affiliations:** Department of Civil and Industrial Engineering, University of Pisa, largo L. Lazzarino, I-56122 Pisa, Italy

## Abstract

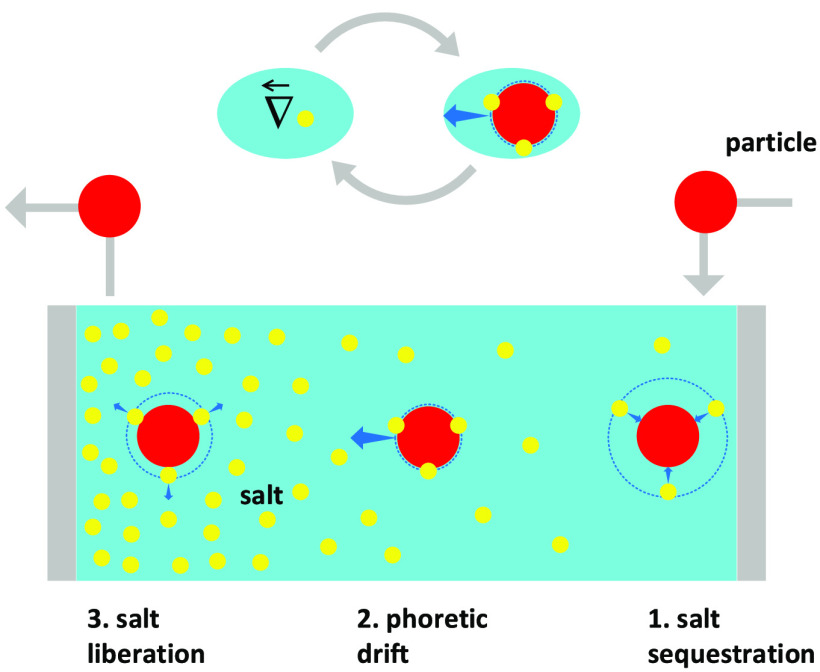

We show that colloidal suspensions that acquire a surface charge by capturing ions from the surrounding solution display unexpected and remarkable phoretic behavior. Depending on suspension volume fraction, a critical zeta potential ζ exists where the effective electrophoretic mobility diverges, becoming virtually infinite. Beyond such critical value, a ζ-range is identified where mobility reversal occurs, i.e., the effective mobility becomes negative. This counterintuitive behavior is due to the salt gradient engendered by phoretic drift of this kind of particles, which capture and release ions (salt), respectively, at the start and the end of the phoretic path. This salt gradient deeply influences the electric field in the bulk electrolyte where the particles migrate: it can make the field vanish, hence the mobility divergence, or even entail inversion of the field, which is reflected in the mobility reversal. These findings should spur new concepts in a variety of traditional and emerging technologies involving, for example, the separation or targeting of colloids as well as in applications where the creation or manipulation of chemical gradients or electric fields in solution is critical.

## Introduction

When a suspension of surface-charged, colloidal particles is subjected to an external electric field, the particles move through the electrolyte solution.^1^ This electrokinetic phenomenon, named electrophoresis, has a variety of important technical applications. These include separation/deposition of biopolymers, drugs, and cells^2^ and electrophoretic coating/painting^3^ (currently used also to form the protective coating layer on car bodywork). Moreover, new applications largely based on this phenomenon are emerging in connection with the development of micro- or nanofluidics^4−6^ and novel, advanced materials/products and technologies.^2,7^ The basic physics of electrophoresis is as follows: in the simplest representation, the colloidal particle in an electrolyte medium has a fixed electric charge distributed on its own surface. According to Boltzmann statistics, that induces a diffuse atmosphere around the particle of free ions bearing opposite charge, usually called the diffuse Debye layer (DDL).^1,8^ In a mean-field representation, the electrolyte medium in the DDL is homogeneously charged. Accordingly, in the presence of an external electric field, it is subject to a body force, which obviously determines relative motion of the liquid medium itself with respect to the oppositely charged particle. As a result, the latter moves with respect to the far liquid supposed at rest in the laboratory reference frame.

The most popular theory of electrophoresis, due to Smoluchowski,^9^ furnishes for a spherical particle in a uniform, external electric field, –Ψ_∞_, the well-known expression of electrophoretic velocity
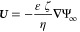
1where ε and η are the dielectric constant and the viscosity of the liquid, ζ is the potential at particle surface relative to far liquid, also termed zeta potential, and Ψ the external electric potential. Subscript ∞ denotes large distance from the particle (in the far bulk electrolyte). Equation 1 is valid in the thin-DDL limit (DDL thickness ≪ particle diameter). The case of thick-DDL limit for a sphere (diameter ≪ DDL thickness) was first addressed by Debye and Huckel^10^ and, later on, further settled by Henry.^11^ Those early theories of electrophoresis have known extensive and important development over several decades. For thin DDL, Morrison^12^ showed that eq 1 holds in general, whatever the particle shape (and size). Moreover, he demonstrated that the hydrodynamic flow field around the particle is irrotational and the particle translates without rotation. DDL relaxation (i.e., distortion of the ion atmosphere in the DDL) as a consequence of an external field was first pointed out by Mooney,^13^ although the modeling of this effect was developed by Overbeek and co-workers^14,15^ and, independently, by Booth.^16^ All those works also predict DDL relaxation to vanish in the thin-DDL limit with recovery of Smoluchowski’s result. Another important line of development of the theory of electrophoresis concerned the effect brought about by excess surface-current density, i.e., the current associated with tangential ion transport in the DDL relative to current in the bulk electrolyte. For a long rod collinear with the applied field, Bikerman^17^ observed that the field intensity at particle surface is attenuated as a result of such surface current, which translates into a drop of the apparent electrophoretic mobility. Later on, Henry^18^ and Booth^19^ independently introduced the surface-conductivity concept in the calculations of the electric field about an electrophoretic sphere. Both these authors predicted a dependence of mobility on sphere radius. However, the influence of the excess ion transport in the DDL was correctly and exhaustively addressed in theoretical works by Dukhin and co-workers published in the Russian literature^20^ and, later on, in the contributions by O’Brien and co-workers.^21,22^ Those authors considered thin DDL and showed that the excess surface transport determines perturbations both of the electric and salt concentration fields around the particle (specifically in the electroneutral solution outside the DDL). In turn, these perturbations bring about a change in the effective electrophoretic mobility. They also showed that these effects become important only for “logarithmically” large zeta potentials, namely, for a binary and symmetric electrolyte: e^*ze*|ζ|/2*k*_B_*T*^ ≫ 1 (*k*_B_, *T*, *z*, and *e* denoting the Boltzmann constant, absolute temperature, anion/cation valence, and elementary charge, respectively). Building on those theories, Tricoli and Orsini^23^ have addressed more recently the electrodiffusiophoresis of a highly charged particle under simultaneously acting salt gradient and electric field—a situation encountered in various technical applications (here, highly charged is used to refer to particles possessing logarithmically large zeta potential). Similar to Dukhin’s and O’Brien’s theories, strong departure from Smoluchowski behavior was predicted due to excess surface transport in the highly charged DDL. Moreover, a number of notable effects were found, the most striking one being perhaps anisotropy of the phoretic drift for non-spherical particles, such as cylinders and spheroids.^24^ Another recent theoretical development concerned the diffusiophoresis of soft (permeable) colloids in electrolyte solution. Interestingly, for sufficiently high charge of the particle, reversal of mobility was predicted over a certain range of ionic strength.^25^

All the above studies considered a single particle in an infinite electrolyte solution. In some cases, their results are readily applicable to phoresis of an ensemble of particles (colloidal suspension) as well. However, as shown for the first time in the present communication, in other cases, new fundamental issues arise for an ensemble of particles depending on the type of charged surface, which cause the vanishing or even the inversion of the electric field in the bulk suspension (−Ψ_∞_) relative to the externally applied field (at the electrodes). These unexpected phenomena entail striking electrophoretic behavior featuring, respectively, enormous effective mobility or, even more surprisingly, mobility reversal.

The remainder of the paper is organized as follows. In the next section, we define the model physical system, in which these new phenomena can manifest and identify the physicochemical characteristics the colloidal particles must possess for these phenomena to occur. We also show how these phenomena are related to a large-scale salt gradient that is self-generated within this system as a result of the phoretic drift itself. In the Methods section, we present the mathematical formulation of the system and obtain the analytical expression of the effective electrophoretic mobility. In the subsequent section, the analytical results are presented and discussed in depth. Finally, we summarize the main points of this work and indicate possible implications and developments.

## Model Physical System

In an electrolyte solution coupled with an electric field, a concentration gradient of the electrolyte (termed salt gradient hereafter for brevity) is, in general, also present.^26^ Therefore, as pointed out earlier,^27^ in an electrophoretic process, a salt gradient interlocked to the electric field may arise. For a binary and symmetric electrolyte where the current is carried by only one ion species (while the other is a spectator ion), the far-field relation between salt gradient and electric field reads^23^
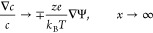
2where *c* denotes numeral salt concentration and ***x*** is a position vector in a suitable reference-frame. Basically, eq 2 expresses the condition that the spectator ion, being unreactive to the electrodes, must stand still at all places in the bulk electrolyte solution (zero-flux condition). Signs “–” and “+” refer to the cases where current in the bulk electrolyte is carried, respectively, by the negative and positive ion. However, even in the situation where only one ion type is reacting at the electrodes, a new far-field condition must be formulated in place of eq 2 for a suspension of particles that acquire (lose) a surface charge by capturing (releasing) ions from (into) the surrounding solution. In fact, these particles carry salt from one boundary of the suspension domain (where they get charged) to another (where they get discharged). Clearly, this process engenders a salt gradient, which has deep impact on the electric field in the bulk suspension, as shown below. Before describing the system, it is worthwhile to point out that electrophoretic particles of the kind specified above are encountered in a very large variety of situations: all particles charged by anion or cation adsorption belong to such class, including gas bubbles and hydrocarbon oil droplets in electrolyte solution.^28,29^ Also included are particles able to reversibly bind ions from the surrounding solution. This is a vast class which, in turn, includes clays and a variety of other types of mineral particles, such as oxide-,^30,31^ silica-, sulfide-, and salt-type minerals.^46^ Other examples are particles possessing nucleophilic surface groups able to capture *H^+^* ions from the surrounding solution at sufficiently low pH. Conversely, the protonated groups release the *H^+^* ions at sufficiently high pH. Included in this class of particles are proteins as well as a variety of functionalized polymers or resins.^28^

**Figure 1:**
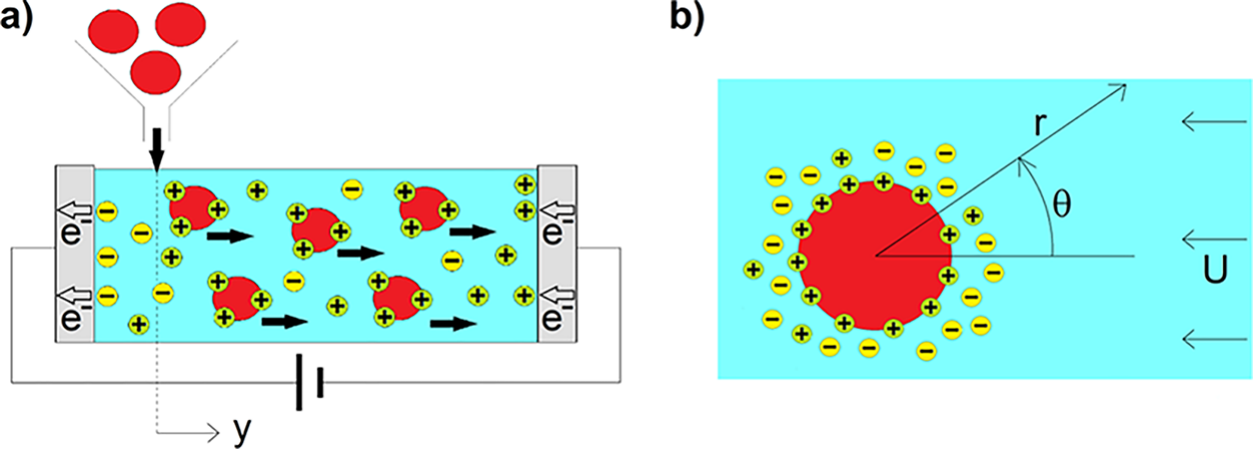
Schematic of the system. (a) As soon as introduced into the electrophoretic bath (at *y* = 0), the electrically neutral particles immobilize a certain amount of cations onto their own surface, thereby acquiring a fixed positive surface charge. (b) A corresponding amount of (negatively charged) counter-ions is also sequestered into a diffuse fluid atmosphere (DDL) surrounding the particles.

As a generic case study, let us consider the model system depicted in Figure 1a and described below. Typically, the external electric field is created in the electrolyte solution by passing an ion current between two electrodes (cathode and anode). The passage of current bears electrochemical reactions at both electrodes of at least one ion species present in solution. For simplicity, we consider a binary and symmetric electrolyte and only one ion species reacting at the electrode. This affords focus just on the novel fundamental issues addressed in the present study while unnecessary algebraic complications are avoided (incidentally, the instance of only one ion species reacting at the electrodes is most common). The system is considered at steady state, whereby electrically neutral particles are continuously fed (at the proper rate) to the solution at a given location (*y* = 0 in Figure 1a) between the electrodes. As soon as introduced into the solution, each particle acquires an electric charge by capturing one ion species from the electrolyte and fixing it onto its own surface while a cloud of oppositely charged ions (counter-ions) from the solution is also sequestered into the DDL (Figure 1b). Without loss of generality, represented in Figure 1 are particles bearing positive surface charge. Then, the charged particles migrate toward an electrode (the cathode in Figure 1) where they lose their charge, coagulate, and, crucially, liberate into the solution both the cations previously immobilized onto the surface and the excess anions of the DDL. It is worth noticing that in this setting, the electrophoretic particles act like salt carriers. Clearly, then, the bulk solution is salt-depleted and salt-enriched where the particles are, respectively, introduced and discharged. As a result, a salt gradient arises on cell length scale, which influences the overall phoretic drift of the particles. Indeed, this influence can be dominant as shown in the Results section. Before getting into the formulation of the problem, it is worthwhile to illustrate a significant real example of the above prototype system that is related to the automobile industry. This is the coating process of car bodywork with a thin, passivating layer of suitable material. There, a coating layer is produced by electrophoretic deposition (cataphoresis) of organic colloidal particles onto the car bodywork, which itself is the cathode electrode.^32^ In that case, the electrolyte is typically acetic acid, and the cathode and anode reactions are, respectively
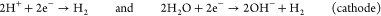
3a
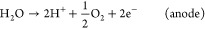
3b

Then, overall, there is hydrogen-ion generation and consumption, respectively, at the anode and the cathode. In the present example, the particles are essentially made out of modified epoxy resin with tethered amine groups. As soon as the particles are added to the electrolyte solution, the amine groups on the surface, due to their nucleophilic character, capture protons by acid–base reaction with acetic acid in solution,^32^ thereby conferring a fixed, positive surface charge upon the particles themselves. Crucially, a corresponding amount of negatively charged acetate counter-ions is also subtracted from the electroneutral bulk solution and sequestered into the DDL. Thus, in this example, protons are the ions captured and immobilized at the surface, while acetate anions are the counter-ions of the charged DDL. Then, the charged particles migrate to the cathode. There, the reverse process (particle discharge) occurs with release of the *H^+^* ions previously captured by the particles; the particles return to their initial, uncharged state and coagulate, thus forming the coating layer.^33^ It should be noted that the portion of *H^+^*-current transported by the particles does have a counterpart in the electrolyte. In fact, upon particle discharge and deposition, a corresponding amount of acetate counter-ions from the disrupted DDL is also liberated into the electroneutral solution. These ions, then, must move away from the cathode by concomitant diffusion and electromigration, thereby contributing to the total electric current and influencing the salt-concentration field in the bulk electrolyte. Thus, although *H^+^* is the sole ion species participating to electrode reactions, it is not the only one carrying the current in the electrolyte. Accordingly, eq 2 is not valid for the type of colloidal systems concerned here. In general, the proper far-field condition relating *c*_∞_ with Ψ_∞_ can still be obtained by integration of the co- or counter-ion conservation equation in the electroneutral domain, taking all electrochemical processes at domain boundaries into due account. To do that, let us refer again to the (unidimensional) system of Figure 1a supposed at a steady state. Also, the particles are supposed to be far away from one another (distance between any two particles suitably larger than particle size), bearing the absence of hydrodynamic interactions between particles (incidentally, as pointed out in the Methods section, the flow field caused by the drift of the particles is irrotational). Under these conditions, the net amount of fluid displaced by each particle is null, which has two important consequences: first, there is no net convection on a length scale suitably larger than the particle size; second, the far fluid is effectively quiescent. It is also assumed for simplicity that the particles are removed from the system as soon as they discharge at the electrode. In this way, there is no net backward flux of fluid volume. This assumption just avoids spurious effects connected to time variation of the position where particle discharge takes place (i.e., moving cell boundary). However, clearly, it does not limit or affect in any way the fundamental physical issues concerned here. Indicating with ***J*** the particle flux density and *N* the excess number of counter-ions sequestered in the DDL of a charged particle, a local balance of total inert (i.e., not reacting at the electrodes) ions on a length scale much larger than particle size provides, at a steady state,
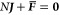
4

In eq 4, *N****J*** represents the flux of spectator ions associated to the phoretic flux of the particles. These are the spectator ions either present in the DDL (if they are the counter-ions) or bound to particle surface (if they are the co-ions). ***F̅*** denotes the averaged flux density of inert ions in the (electroneutral) fluid domain external to the DDL, the average being performed over a fluid domain that is large enough to enclose many particles and, at the same time, much smaller than the size of the electrophoretic cell. In deriving eq 4, the physical condition of zero flux of the inert ion species at all boundaries of the cell has been used. Equation 4 is valid in general regardless of whether the inert ion species is either the one sequestered into the DDL (counter-ion) or the one immobilized at particle surface (co-ion). Of course, the expression of ***F̅*** changes for either case. Denoted by *Z*, the volume fraction of the particles in the suspension, analytical expressions for ***F̅*** correct to *O*(*Z*) are derived in Appendix A for a statistically isotropic suspension, assuming that *Z* is small enough to make all interactions between any two particles negligible. Not surprisingly, in both cases, ***F̅*** results as proper linear combinations of the unperturbed salt and potential gradients  and Ψ_∞_ (hereafter, subscript ∞ denotes distance (from the particle), which is large enough as compared with particle size (whereby the concentration and electric fields are unperturbed by the particle itself) and, at the same time, small as compared with the size of the electrophoretic cell). Also, it is important to point out, for a statistically isotropic suspension of dilute (non-interacting) particles, that the field surrounding each particle is same as for the one-particle problem (i.e., single particle in infinite space) with far-distance field uniform and equal to the average field in the suspension, the average being calculated over a volume containing a large number of particles, which nevertheless are a small portion of the whole suspension.^34,35^ Thus,  and Ψ_∞_ are, in fact, the volume-averaged salt and potential gradients.

Equation 4 is effectively the far-field condition interlocking *c*_∞_ and Ψ_∞_. It is more general than eq 2 as it also involves explicitly the electrophoretic particle flux ***J***. For a single particle, whereby particle concentration is virtually null and ***J*** vanishes, eq 4 automatically reduces to eq 2. For an ensemble of particles (colloidal suspension), eq 4 also reduces to eq 2 when the inert ions stand still in the (electroneutral) solution external to the DDL on length scale of the electrophoretic cell. That occurs for particles not acting as salt carriers, i.e., particles that undergo mere ion dissociation, without co-ion immobilization upon immersion in the solution. Otherwise, eq 4 applies as such. Shown in the next sections is how this condition produces new, striking effects on the electrophoretic behavior for colloidal suspensions with sufficiently high surface charge.

## Methods

Here, we obtain the analytical expression for the effective electrophoretic mobility of the colloidal suspensions of the type described in the previous section. The basic steps of the procedure involve the formulation of the governing equations for the electric potential and salt concentration and the pertaining boundary conditions. The solution of this differential problem allows the determination of the hydrodynamic flow field around the particles, which preludes to the analytical calculation of the effective phoretic mobility.

Before starting the analysis, it is convenient to summarize the basic hypotheses. First, we consider thin DDL. To enable sufficient analytical progress, we assume negligible interactions of the hydrodynamic, electric, and salt concentration fields between particles, which holds true when the volume fraction of the particle suspension is sufficiently small. This assumption will be revisited in the Discussion section. Moreover, the suspension is assumed to be statistically isotropic. To best focus on the new effects stemming from eq 4, we keep the analysis as simple as possible by considering spherical particles (of radius *R*), binary and symmetric electrolyte (of generic valence *z*), and weak applied field in the sense that will be specified later. Finally, the system is considered at a steady state.

The slip velocity at particle surface due to the simultaneous actions of salt and electrical potential surface gradients was addressed by several investigators, assuming ideal behavior of ions in solution (i.e., only electrostatic interactions).^20,22−24,36,37^ It results not only from the electro-osmotic flow in the DDL due to the tangential electric field (like in Smoluchowski’s theory) but also from the diffusio-osmotic flow due to the tangential (osmotic) pressure gradient in the DDL caused in turn by the salt-concentration gradient. The surface-slip velocity can be expressed in the form^23^
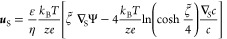
5

Symbol ^∼^ denotes normalization with respect to the thermal voltage *k*_B_*T*/*ze*, whereas  denotes the surface differential operator. Hereafter, “surface” is intended at the outer edge of the particle DDL. Moreover, for weak applied field, it was shown that both the electric potential and salt concentration obey the Laplace equation in the bulk, electroneutral domain (outside the DDL):^20,22,23^
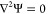
6
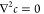
7

The effective boundary conditions for eqs 6 and 7 at particle surface (which account for tangential ion transport in the DDL) were formulated by several authors.^20,22,23,38^ Here, they are presented in the form
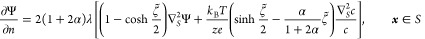
8
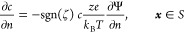
9where *n* is the coordinate perpendicular to the surface and facing outward, α = ε(*k*_B_*T*/*ze*)^2^/η*D* is the (non-dimensional) ion-drag coefficient, and λ is the Debye length, expressed for a binary and symmetric electrolyte as . For simplicity, we have supposed the same diffusivity *D* for anions and cations. Full details about the derivation of boundary conditions 8 and 9 can be found in ref (23). Finally, the differential problem is closed with the two far-field conditions
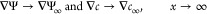
10

It is worth noting in eq 10 that while Ψ_∞_ is typically intended as assigned, *c*_∞_ is unknown at the moment. However, it will be determined (along with the electrophoretic velocity or particle flux ***J***) by imposing eq 4. Also, *c*_∞_ will turn out to be a constant interlocked to Ψ_∞_. It is important to mention that *c*, although varying in the bulk domain around the particle, is constant (equal to *c*_∞_) at leading order for weak applied field. This fact enables considerable analytical progress because the surface eqs 5, 8 and 9 also become linear. The weak-field linearization will be illustrated in detail and discussed further below in the Discussion section.

In brief, the procedure to arrive at the effective electrophoretic mobility is as follows: first, the above differential problem is solved for Ψ and *c*, thereby calculating the analytical expressions for Ψ and *c*. These are then substituted into eq 5 to obtain the analytical expression of the surface-slip velocity. As illustrated further below, the latter affords full determination of the flow field around the particle in terms of Ψ_∞_ and *c*_∞_. Finally, eq 4 is enforced to obtain the particle velocity in terms of the sole assigned field Ψ_∞_, hence the effective mobility.

With regard to an individual particle, the problem is referred to spherical coordinates as defined in Figure 1b, with the polar axis chosen oriented along Ψ_∞_. Given the one-dimensionality of the cell, both vectors *c*_∞_ and ***J*** are clearly parallel to Ψ_∞_. However, as proven in Appendix B, the three vectors are always parallel also for arbitrarily shaped bi- or three-dimensional systems. Thus, the following derivation applies in general. Solutions of eqs 6 and 7 satisfying far-field condition 10 have the general form:
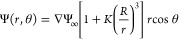
11
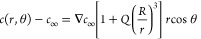
12

Scalars ∇Ψ_∞_ and ∇*c*_∞_ are, respectively, the coordinates of Ψ_∞_, *c*_∞_ on the polar axis. While ∇Ψ_∞_ is (by definition) always positive, ∇*c*_∞_ can be either positive or negative (*c*_∞_ having same or opposite orientation with respect to Ψ_∞_) depending on the circumstance. *K* and *Q* are (as yet undetermined) non-dimensional constants. These are calculated by subjecting eqs 11 and 12 to the surface boundary conditions 8 and 9. Then, *K* and *Q* are conveniently expressed as follows:
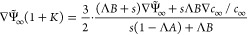
13
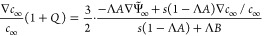
14where *A* = 1 – cosh (/2), *B* = sinh (/2) – α/(1 + 2α), and Λ = 2(1 + 2α)λ/*R*, whereas *s* stands for sgn(ζ). The tangential gradients (at particle surface) of electric potential and concentration are, respectively,
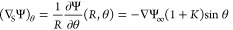
15
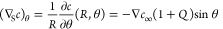
16

Then, substituting eqs 13 and 14 into eqs 15 and 16, respectively, and subsequently substituting the latter into eq 5, we obtain the expression of the surface-slip velocity:
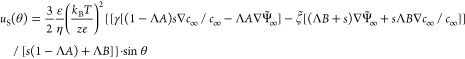
17with γ = 4ln[cosh(/4)]. Crucially, as noted, *u*_S_ is proportional to sin θ, which bears that the flow field around the particle (outside the DDL) is one of potential flow (whereby the force-free and torque-free conditions are automatically satisfied). Accordingly, the electrophoretic velocity *U* of the particle is immediately determined by the expression^39^
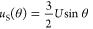
18

Comparing eq 17 with eq 18, we obtain the following expression of the electrophoretic velocity:
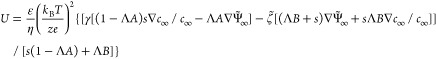
19

It should be borne in mind that ∇*c*_∞_ is as yet unknown. Equation 4 (after suitable manipulation) is now going to be imposed to express *c*_∞_ in terms of Ψ_∞_ and ***U***. We first proceed to obtaining useful expressions for *N* (the number of excess counter ions sequestered in the DDL, i.e., the amount of salt captured and carried by each particle), ***J*** (the flux of particles per unit area), and ***F̅*** (the averaged flux density of inert ions in the (electroneutral) fluid domain external to the DDL) to be inserted in eq 4. *N* is calculated just by integrating the excess counter-ion concentration along the DDL thickness:
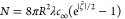
20

Regarding ***J***, with *C*_P_ being the numeral concentration of the particles, we have
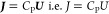
21

The calculation of ***F̅*** is somewhat involved and is reported in Appendix A. As it turns out, ***F̅*** is expressed as either
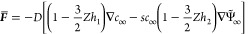
22aor
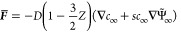
22bdepending on whether the spectator (inert) ion species is, respectively, the counter- or co-ion in the DDL. The expressions of *h*_1_ and *h*_2_ in eq 22a are *h*_1_ = (1 – Λ*A* – sΛ*B*)/(1 – Λ*A* + *s*Λ*B*) and *h*_2_ = (1 + Λ*A* + sΛ*B*)/(1 – Λ*A* + *s*Λ*B*). It is worthwhile to make some comments on eqs 22a and 22b, bearing in mind that ***F̅*** is the volume average of the inert-ion flux in the region external to the particle DDLs. We start with eq 22b: this is formally identical to the well-known Maxwell formula for macroscopic conduction/diffusion through a matrix of a dilute random dispersion of impermeable spheres. The reason is that in this case, the spectator ion is the co-ion, which does not cross the boundary separating the DDL from the external domain (cf. eqs A1, A2b, and 9). Thus, each particle, considered as including its own DDL, is effectively impermeable to the spectator–ion flux, where the classical Maxwell result for insulated spheres is recovered. The situation is different with eq 22a. Here, the spectator ion is the counter-ion, which clearly crosses the external boundary of the DDL (cf. eqs A1, A2a, and 9). Thus, the particle (inclusive of the adjacent DDL) is not impermeable, and that results in the expression 22a for ***F̅***.

We now plug eqs 21 and 22a or 22b into eq 4 to respectively obtain
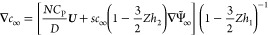
23aor
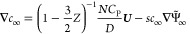
23bwith *N* expressed by eq 20. Finally, substituting eq 23 into eq 19 and noting that *C*_P_ (the numeral concentration of the particles) is related to *Z* (the volume fraction of the particles) by *Z*, we arrive, after rearranging, at the electrophoretic velocity of the particles
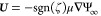
24with μ, the effective electrophoretic mobility, expressed as either
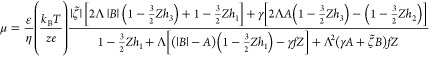
25aor
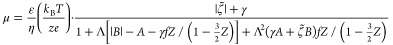
25bwhere *h*_3_ = (*h*_1_ + *h*_2_)/2 = (1 – Λ*A* + *s*Λ*B*)^−1^ and *f* = (3α/1 + 2α)(*e*^|ζ̃|/2^ – 1). Again, either eqs 25a or 25b applies when the spectator (inert) ion species is, respectively, the counter- or co-ion in the DDL.

According to eq 23, ∇*c*_∞_ results as a linear combination of *U* and ∇Ψ_∞_. Then, substituting into eq 19, we are left with an equation of the form
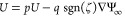
26with *p* and *q* as non-dimensional and dimensional coefficients, respectively, which are known functions of , λ/*R*, *Z*, and α. This form explicates the effect of the cell-scale salt gradient engendered by the phoretic drift. This effect is represented by the *pU* term in eq 26, and its relative importance is measured by the magnitude of the (non-dimensional) coefficient *p*: small (or null) *p* corresponds to a negligible (or absent) effect. Possibly, finite values of *p* correspond to interesting situations. In this regard, it is convenient rearranging eq 26 into the form
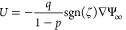
27

While *q* is always positive (cf. eq 27 with eqs 24 and 25), generally, it is *p* < 1, so that the mobility is positive. However, mainly depending on , λ/*R* and *Z*, it may be *p* > 1, in which case mobility reversal occurs. Moreover, for *p* ≅ 1, the magnitude of the mobility becomes very large, diverging at *p* = 1, where the denominator vanishes. A detailed account of this anomalous behavior is given in the Results section, while the physics underlying the singularity and reversal of the mobility is addressed in the Discussion section.

## Results and Discussion

### Results

**Figure 2:**
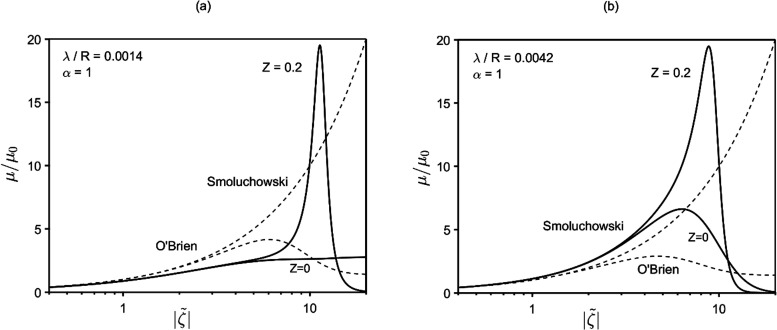
Mobility vs zeta potential as a function of particle size and concentration. In the small- regime Smoluchowski behavior is recovered. Panels (a) and (b) reflect eqs 25a and 25b, respectively.

**Figure 3:**
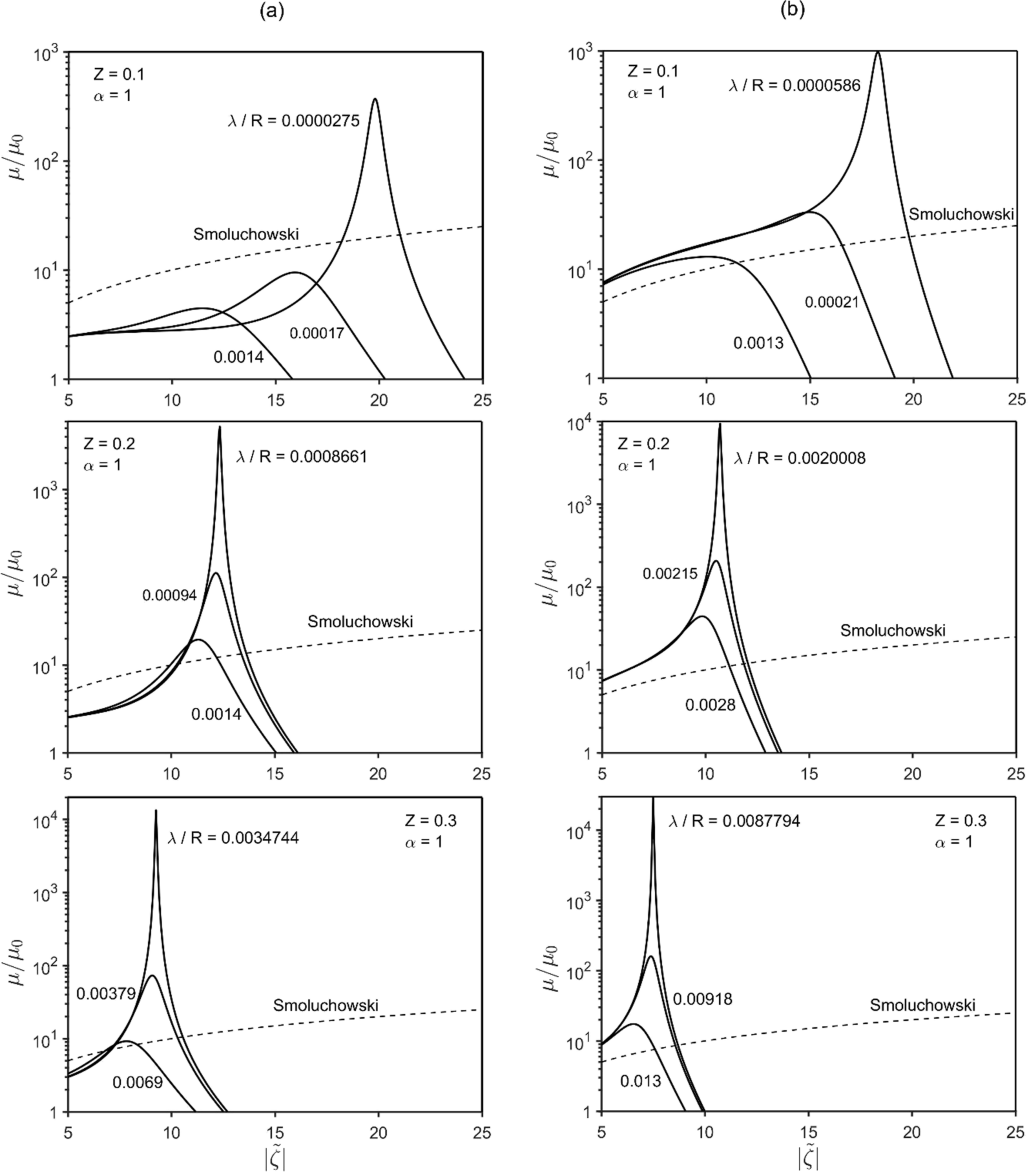
Mobility vs zeta potential as a function of λ/*R* and particle concentration (*Z*). Within certain ranges of zeta potential, the mobility can be enormously (orders of magnitude) larger than the classical Smoluchowski mobility. Panels (a) and (b) reflect eqs 25a and 25b, respectively.

**Figure 4:**
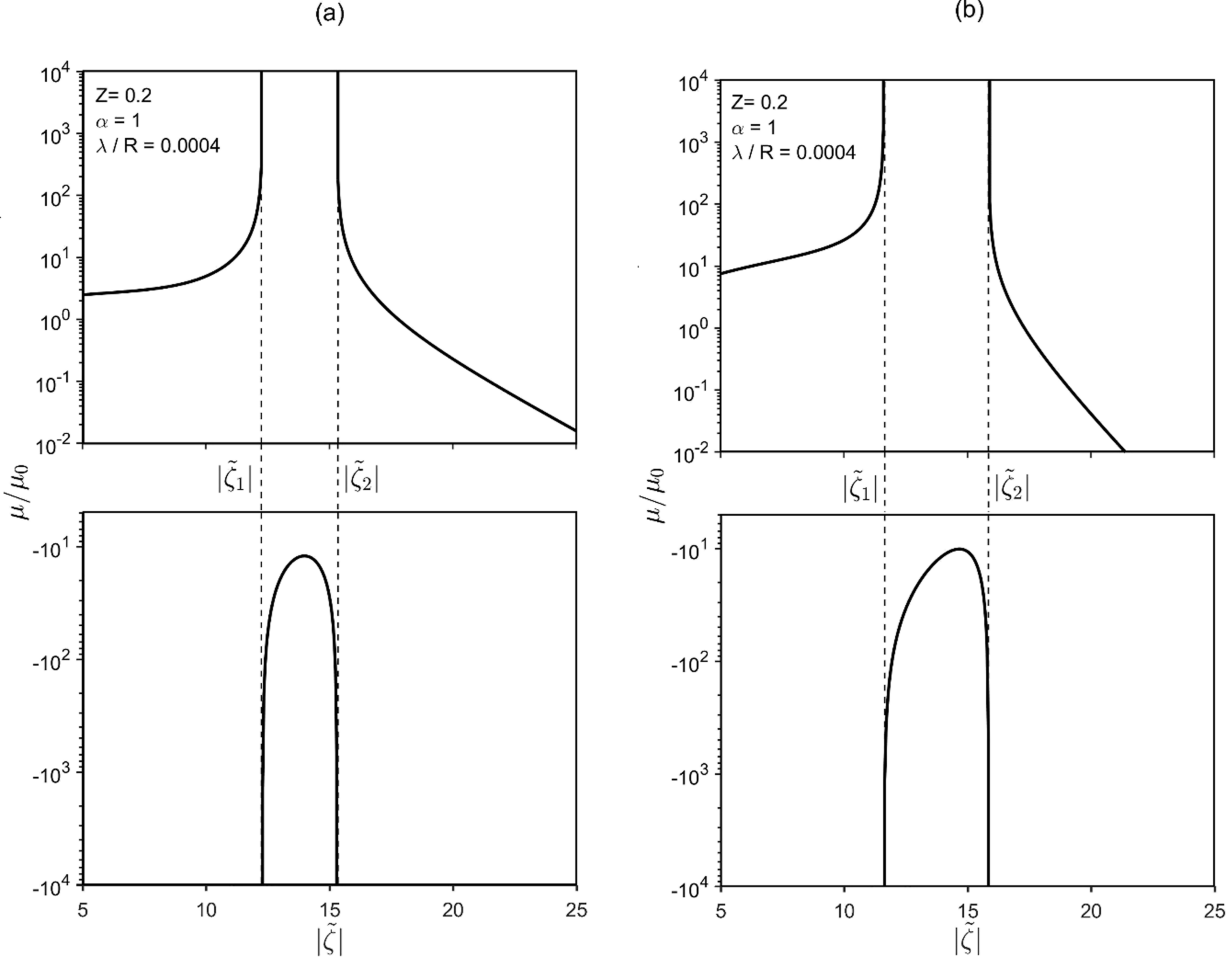
Mobility vs zeta potential: within certain *Z* and λ/*R* ranges, a  interval exists wherein mobility is negative. Also, at the two extremes of this interval, the mobility diverges. Panels (a) and (b) reflect eqs 25a and 25b, respectively.

**Figure 5:**
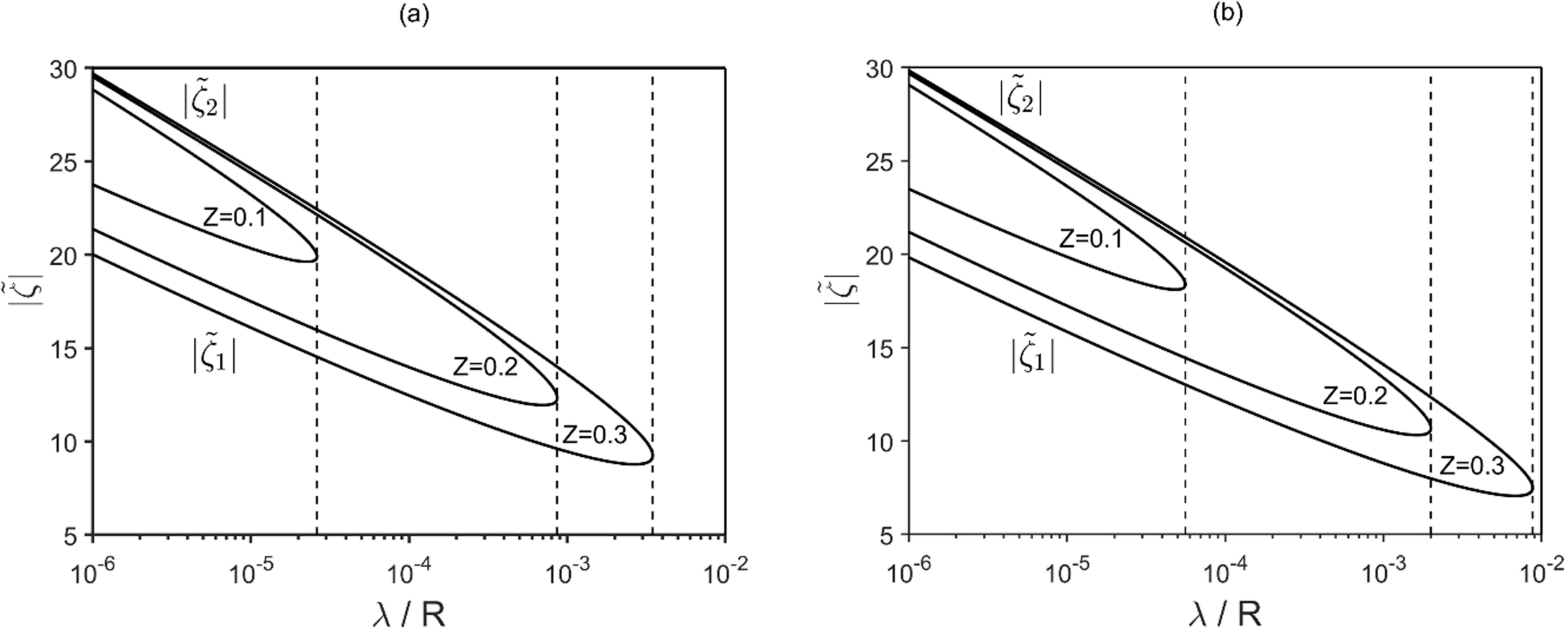
Plot of  and  as a function of λ/*R* for various *Z*. Mobility reversal occurs between  and . Panels (a) and (b) reflect eqs 25a and 25b, respectively.

**Figure 6:**
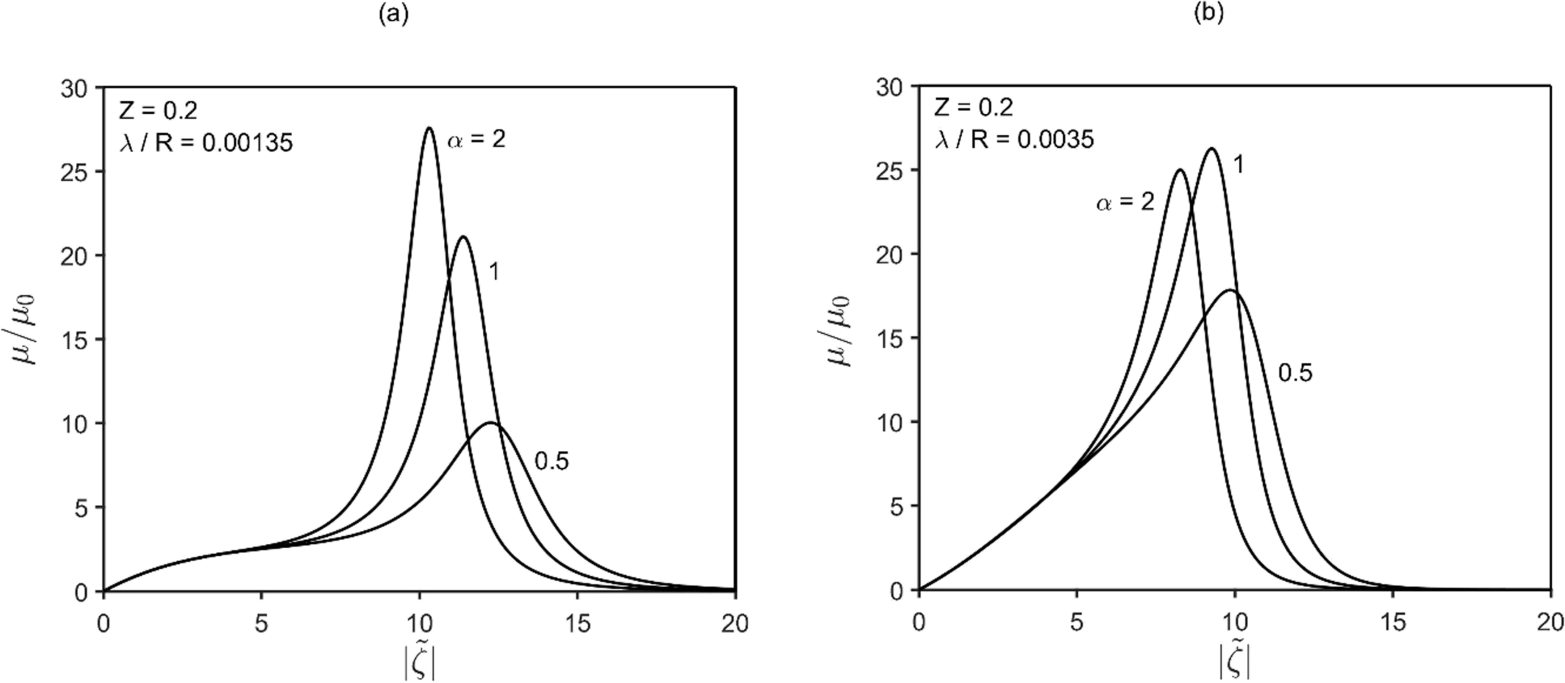
Influence of the ion-drag coefficient *α* on the mobility. Panels (a) and (b) reflect eqs 25a and 25b, respectively.

**Figure 7:**
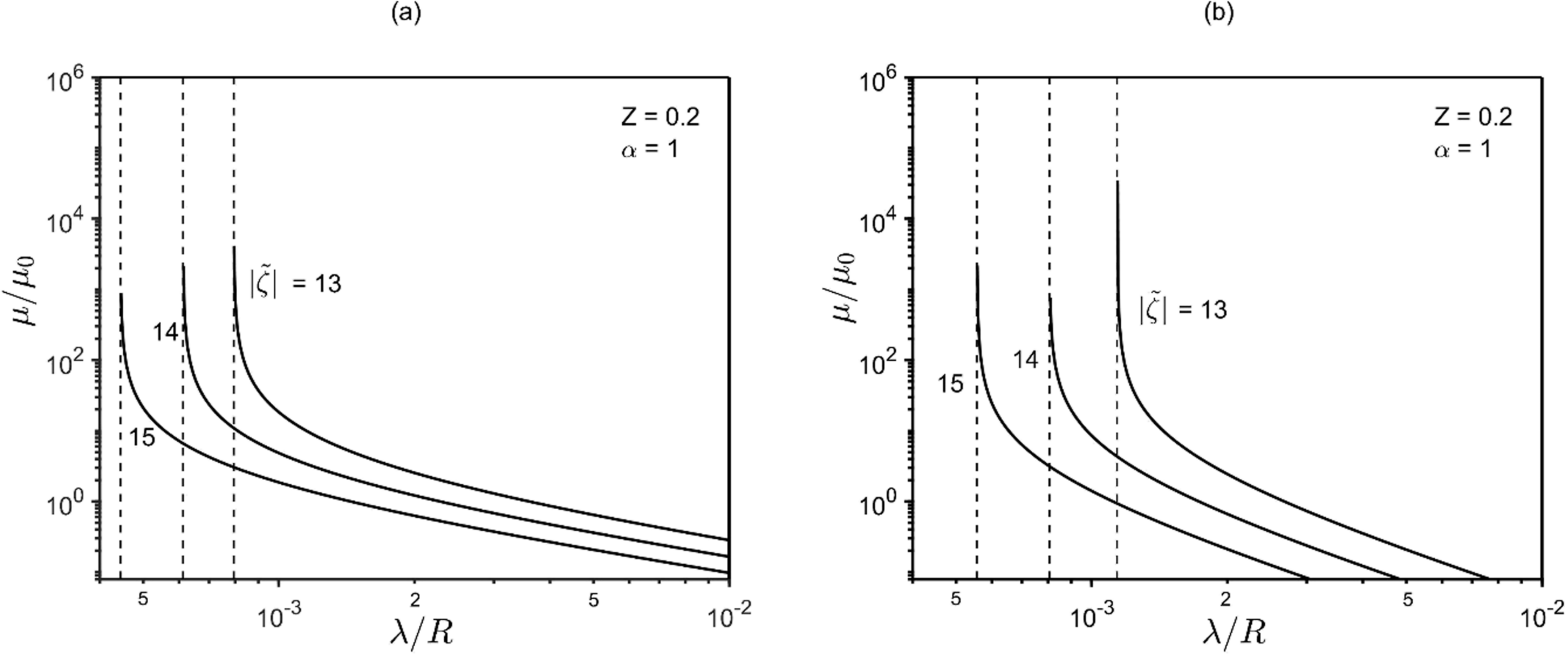
Mobility vs λ/*R* for various zeta potentials. On the left part of the plots an increasing sensitivity of mobility to λ/*R* is observed. Panels (a) and (b) reflect eqs 25a and 25b, respectively.

Figures 2–7 show the plots of the non-dimensional mobility (scaled to μ_0_ = (ε/η)(*k*_B_*T*/*ze*)) as a function of , λ/*R*, *Z* and α. The plots denoted by (a) and (b) reflect eqs 25a and 25b, respectively. The Smoluchowski mobility (ε/η) scaled to μ_0_ is also plotted for comparison. It is worthwhile noting in eq 25 that *h*_1_, *h*_2_, *h*_3_, *A*, and γ are all even functions of , while *B* is odd. Then, μ as a function of  is also even. As expected, in the small- limit, wherein the amount of salt carried by the particles is negligible and the surface-current effects also vanish, the mobility duly complies with Smoluchowski law (eq 1), independent of λ/*R*, *Z*, and α (Figure 2). Also shown in Figure 2 are the plots for *Z* = 0 and the plots of O’Brien,^22^ both valid for a single particle in an infinite electrolyte medium, whereby the cell-scale salt displacement by the phoretic motion of the particle is null (the difference between the two is that while the plots of O’Brien consider null far-field salt gradient, the *Z* = 0 plots account for the far-field salt gradient interlocked to the far electric field as from eq 2). Clearly, for *Z* > 0 (*Z* = 0.2 in Figure 2), the effect of the salt gradient engendered by the cell-scale salt displacement associated to the collective phoretic drift of the particle suspension is sensible. This effect can be very strong for suitable values of the parameters, giving rise to remarkable anomalies of the phoretic behavior as shown further below.

Depending on λ/*R* and *Z* (here, the plots refer to α = 1), the mobility displays in general non-monotonous behavior as a function of  with a maximum. As observed in Figure 3, the maximum can be very peaked for sufficiently large *Z* and small λ/*R*, with μ becoming enormously larger than the Smoluchowski mobility in a certain  range.

Depending on *Z*, a critical value of λ/*R* exists, below which the mobility-vs- behavior becomes even more anomalous. An example is shown in Figure 4, where the mobility displays vertical asymptotes at two distinct values of zeta potential,  and , diverging respectively to ±∞ and ∓∞. Between such asymptotes, the mobility is finite and negative. The physics underlying negative mobility is addressed in the Discussion section.

Figure 5 plots  and  vs λ/*R* for various *Z* and α = 1. It is worthwhile noticing that  can assume values as low as 7.

The influence of the ion-drag coefficient α on the mobility is shown in Figure 6. Considering that α ranges on values of order 1, its influence on the mobility appears to be relatively moderate.

Figure 7 presents μ-vs-λ/*R* plots for various  and fixed *Z*. On the right side of the (log–log) plots, it is noted that the mobility is asymptotically proportional to (λ/*R*)^−1^ for eq 25a or (λ/*R*)^−2^ for eq 25b. However, as λ/*R* drops, the dependence becomes more and more marked, with the mobility becoming very sensitive to λ/*R*. Eventually, the curves display vertical asymptote with divergence of the mobility, in line with the results of Figure 4.

Turning back to the mobility-vs- behavior displayed in Figures 2–4, for sufficiently large , in all cases, the mobility decreases exponentially to zero as μ/μ_0_ ∼ e^–|ζ̃|/2^/3α(λ/*R*) for eq 25a or μ/μ_0_ ∼ 4e^–|ζ̃|^(1 + 2α)/3α(λ/*R*)^2^ for eq 25b. This is the well-known Dukhin effect, typical of highly charged particles due to the large counter-ion surface current within the DDL.^20−23^ This current produces locally a strong polarization both of electric potential and salt concentration around a particle (outside the DDL). Such polarization always acts in opposition to the phoretic drift, thereby reducing the mobility. Apparently, then, at sufficiently large , these polarization effects become dominant.

### Discussion

We have seen in the Model Physical System section how particles of the type considered carry salts from one boundary of the colloidal suspension to another, thereby producing a salt gradient *c*_∞_, and how this gradient affects the phoretic drift itself. It has been shown in the Methods and Results sections that these effects can involve surprising behavior with divergence or reversal of mobility. Here, an explanation of the physics underlying these singularities is in order. Looking at eq 23, we note that particle velocity *U* remains finite for any finite value of the fields ∇Ψ_∞_ and ∇*c*_∞_. Therefore, according to eq 27, the divergence of mobility for *p* = 1 must entail the vanishing of electric field, not the divergence of phoretic velocity. Also, a negative electrophoretic mobility bears that Ψ_∞_ and *c*_∞_ are antagonistic, with the second prevailing over the other. It should be pointed out that neither Ψ_∞_ nor *c*_∞_ are externally applied driving forces (i.e., directly imposed from the outside). They are just the fields that automatically form in the domain of the suspension upon applying a given external driving force. The two following examples illustrate how this driving force can be a voltage (or a current) imposed between two electrodes or (and) a salt concentration difference externally imposed at two opposite boundaries of the cell or even other than those. Remarkably, when the driving force is an imposed voltage between two electrodes, it is shown how a negative mobility bears an inversion of the electric field, i.e., the field in the suspension domain, –Ψ_∞_, is opposed the voltage externally applied at the electrodes. However, that does not involve a reversal of the particle velocity.

In the first example, we consider a classical situation where the externally applied driving force is a potential difference between the anode and cathode electrodes of the electrophoretic cell. To illustrate the concept, we refer to the cataphoretic deposition process mentioned in the Model Physical System section and portrayed in Figure 1, where the particles first acquire positive charge upon immersion into a sufficiently acidic electrolyte solution by immobilizing hydrogen ions onto the surface thanks to tethered nucleophilic groups.^35^ Then, they travel to the cathode by phoretic drift. At the cathode, there is hydrogen ion consumption/hydroxyl ions generation by electrochemical reaction 3a. Crucially, hydrogen-ions consumption engenders the formation of a *H*^+^-depleted concentration boundary layer in the electrolyte solution nearby the cathode^33^ (not to be confused with the charged cathode DDL). Due to lack of *H*^+^, the particles liberate the immobilized hydrogen ions (and also the DDL counter-ions) upon entering this boundary layer.^33^ As a result they return to the uncharged state and coagulate. As explained in the Model Physical System section, the liberation of the spectator (i.e., not reacting at the cathode) counter-ions bears electrolyte build-up also in the bulk suspension external to the *H*^+^-depleted boundary layer, thereby forming a salt gradient *c*_∞_ in the bulk suspension. Eventually, the system reaches a steady state, in which a bulk salt gradient is established on the cell length scale. This gradient has a two-fold effect: on one hand, it keeps driving (by diffusiophoresis) the particles to the cathode, where salt concentration is highest. On the other hand, it also influences the electric field in the bulk electrolyte domain crossed by the particles, because Ψ_∞_ is interlocked with *c*_∞_ and the particle flux ***J*** (=*C*_P_***U***) by eqs 23a and 23b. It is important to note that in this setting, particle drift is always concordant with the salt gradient, i.e., the (positively charged) particles move toward the cathode regardless of whether the mobility is positive, null, or negative. Accordingly, when (depending on parameters *Z*_,_ λ/*R* and ) the mobility assumes a negative value the electric field in the suspension –Ψ_∞_ must be opposed to the externally applied voltage at the electrodes, meaning that an electric-field reversal occurs within the suspension domain with respect to the cell region outside the suspension. In this situation, the phoretic drift (toward the cathode) is obviously driven by the salt gradient in the suspension *c*_∞_, while Ψ_∞_ resists the motion. In summary, for particle suspensions of the type under consideration, the phoretic drift creates a salt gradient that has a profound influence on both the drift itself and on the electric field Ψ_∞_ that is actually established in the suspension. This influence can cause Ψ_∞_ to be very small (or even vanish), resulting in enormous (or even diverging) electrophoretic mobility. It can also cause the reversal of Ψ_∞_ with respect to the external voltage applied to the electrodes, resulting in negative electrophoretic mobility. Finally, it is important to point out that the vanishing or reversal of Ψ_∞_ depends only on the physical characteristics of the suspension, namely, the parameters *Z*_,_ λ/*R*,  and α, not on the sort of externally-imposed driving force or its intensity. We have noted earlier that a concentration boundary layer forms between the bulk of the suspension and the cathode surface. At the opposite end of the suspension, where the original particles are introduced into the system, another concentration boundary layer is formed, resulting from the balance between co- and counter-ions consumption by the introduced particles and the co- and counter-ions supply from the anode electrode and the other boundary layer, respectively. We note that an electric-potential boundary layer automatically corresponds to a concentration boundary layer. Since negative mobility entails inverted electric field in the bulk suspension, we observe that the electric field at the two opposite ends of the concentration- and potential boundary layer near the cathode has opposite signs, being (by continuity) positive on the side adjacent to the suspension and negative on the side adjacent to cathode surface. Consequently, the electric potential profile must exhibit a maximum within this boundary layer. Similarly, it can be understood that there must be a minimum of the electric potential in the other boundary layer (where the particles are introduced).

The second example shows how the externally imposed driving force that engenders Ψ_∞_ and/or *c*_∞_ can be other than a mere potential or salt concentration difference suitably applied at two separate locations in the system. In particular, we consider a unidimensional system similar to the one of Figure 1, where the particles are gas bubbles or hydrocarbon-oil droplets of suitably small size. They are introduced at one end of the cell containing an electrolyte solution. No electrodes are present. To make the point more direct, we assume the absence of gravity (or neglect the buoyant force of the particles). It is known that gas bubbles and hydrocarbon-oil drops are able to immobilize (by physical adsorption) anions from the solution onto the surface,^28,29^ while a corresponding amount of cations are sequestered into the DDL. Clearly, as soon as particle feeding begins, there is a salt depletion in the bulk solution where the particles are introduced, while the salt concentration is unchanged in the far region. Accordingly, a salt gradient arises on cell scale, which drives the particles to the other end of the cell where the salt concentration is highest. Particles accumulate and subsequently coalesce at this end of the cell. As a result of particle coalescence, a net diminishment of the total interfacial area of the suspension with release of the immobilized anions (and also DDL cations) takes place there along with a salt concentration buildup. Again, we have a system where the phoretic drift of the particles plays an essential role into the salt gradient buildup and, at the same time, the salt gradient effectively drives particle drift. Thus, the process is self-sustaining and eventually reaches a steady state. Moreover, according to eq 4, i.e., eq 23, an electric field also arises in the bulk electrolyte at the cell scale. Since there is no externally applied voltage (no electrodes are present), a question arises as to what is the ultimate, external driving-force which sustains the process as a whole. Apparently, that resides in the difference between the interfacial energy (i.e., surface tension) required to form the bubbles (or the oil droplets) in the electrolyte solution at low salt concentration and the interfacial energy liberated upon bubble (droplet) coalescence in the solution at high salt concentrations. Incidentally, due to the electric field engendered by the phoretic process, this system can deliver electrical energy.

The basic assumptions of the model are listed at the beginning of the Methods section. Here, their validity is discussed. We start with qualifying the weak-field condition. First, the validity of eq 7 is subordinate to the condition of negligible convection:^23^***u***·*c* ≪ *D*∇^2^*c*, where ***u*** is fluid velocity. Since ***u*** ∼ *O*(*U*), such condition assumes the form
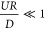
28

The group on the *lhs* is the familiar Péclet number. Second, the validity of eq 6 is subordinate to the condition^23^ It can be shown by scaling analysis that for at-least-logarithmically large zeta potentials (i.e., (λ/*R*) exp(/2) ≥ *O*(1)), it is Ψ · (*c*/*c*) ∼ *O*[(λ/*R*)^2^e^|ζ̃|^*Z* (μ*R*/*D*) ∇Ψ_∞_]∇^2^Ψ, which, in conjunction with eq 24, yields the condition for the validity of eq 6:
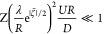
29

The *lhs* of this expression is composed of three non-dimensional quantities: the particle volume fraction *Z*, the Péclet number mentioned above, and the group (λ/*R*)e^|ζ̃|/2^. The latter, apart from an inherently *O*(1) factor, is the well-known Dukhin number^20^ and represents the scale of the ratio of the surface current (flowing in the DDL) to the current flowing in the solution outside the DDL on the particle-size length scale.

Both conditions 28 and 29 must be satisfied. In general, depending on λ/*R*, *Z*, and , condition 29 can be more or less restrictive than condition 28. However, for the values of those parameters concerned by the present study, the two conditions are pretty much equivalent. Moreover, it can be shown by scaling analysis that
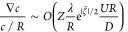
30which, in view of condition 29, implies that ∇*c* ≪ *c*/*R*. Thus, the weak field condition also ensures that the salt concentration around the particle is constant at leading order and equal to *c*_∞_ as assumed in the Methods section.

Another simplifying assumption made in the Methods section is considering each particle of the suspension as surrounded by infinite fluid, equivalent to infinitely dilute particles: *Z* ≪ 1. However, according to eqs 11 and 12, the ∇Ψ and ∇*c* perturbations due to the presence of a particle both decay as ∼(*r*/*R*)^−3^. Moreover, because irrotational flow applies, the velocity perturbation also vanishes as ∼(*r*/*R*)^−3^. This indicates that interaction between particles with thin DDL is negligible when their surfaces are more than ≃1.5 particle diameters far apart, also in accordance with previous detailed analytical study by Reed and Morrison.^40^ Thus, importantly, the theory holds for moderately dilute particles as well.

It is observed in Figures 3 and 4 that for the considered particle concentration values, the peak (or the vertical asymptotes) of the mobility occurs at relatively large zeta potential ( ≥ 7 ÷ 9). Thus, a question arises as to whether such  values can be realistic. In this regard, for a binary and symmetric electrolyte the zeta potential is related to the surface charge density σ by the well-known expression
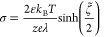
31

Clearly, for a given surface charge, the zeta potential can be increased by increasing the Debye length λ (which in practice can be done by decreasing the salt concentration *c*_∞_). Apparently, then, one could think of employing suitably small *c*_∞_ to accomplish high enough . However, there are lower and upper bounds, respectively, to ion concentration and the Debye length. In fact, the natural dissociation of water imposes that *c*_∞_ can be no less than 10^–7^ per liter (with  denoting the Avogadro number). On the other hand, as noted in Figure 3, the mobility peaks take place at sufficiently small λ/*R* (besides large ), implying obvious superior limitations to λ. Then, the point is whether, for realistic values of surface charge, suitable λ (i.e., *c*_∞_) ranges may exist for the strong effects of Figures 3 and 4 to occur. To address this point, upper limits to realistic surface charge are to be considered first. In this regard, a charge density of one elementary charge per 0.5 nm^2^ (corresponding to 0.3 C/m^2^) is considered typical of a fully ionized surface.^8^ It was also pointed out that such σ value is < *e*/*l*_B_^2^ , with *l*_B_ being the Bjerrum length (≃0.7 nm for univalent ions), which ensures no immobilization or orientation of water dipoles at the charged surface.^8^ Using eq 31, a sample calculation for σ = 0.3 C/m^2^ and salt concentration of 10^–5^ molar furnishes  ≃ 15 and λ ≃ 97 nm, or λ ≃ 48 nm, respectively for univalent or divalent ions. Considering particles of ≃50 μm size, these Debye lengths respectively corresponds to λ/*R* ≃ 0.00388 and λ/*R* ≃ 0.00194. Similarly, for 10^–3^ molar salt concentration and particles of ≃2 μm size, we have  ≃ 9.7 and λ/*R* ≃ 0.0097 or λ/*R* ≃ 0.00484, respectively, for univalent or divalent ions. The above  and λ/*R* values appear compatible with the manifestation of the strong effects observed in Figures 3–5. Finally, it is worth explicitly highlighting the influence of the ion valence *z*. In this regard, it should be borne in mind that  is the zeta potential scaled to *k*_B_*T*/*ze*. Thus, considering, for example, a divalent (symmetric) electrolyte, a  of, say, 9 actually corresponds to a zeta potential of 4.5*k*_B_*T*/*e* (≃115 mV), a practically viable value. In addition, it should be borne in mind that the Debye length is inversely proportional to *z*. As noted in Figures 3, 5, and 7, lowering the Debye length (λ/*R*) amplifies phoretic mobility, eventually leading to the divergence and inversion of it. In short, the use of higher-valence electrolytes makes more accessible the conditions under which the anomalies observed in Figures 3 and 4 can be realized in practice.

As from Figures 3 and 5, for volume fraction of particles less than 0.3 (0.1 ≤ *Z* ≤ 0.3), mobility behavior is most anomalous for  ≳ 7 ÷ 9. Importantly, also noted in the figures is a downward shift of such  interval as *Z* increases. Thus, it would be interesting to see if the anomalies featured in Figures 3 and 4 can be encountered at more moderate (and common) values of the zeta potential for suitably concentrated suspensions. It should be recalled that the present analysis is valid only for sufficiently dilute suspensions. However, the following physical considerations afford some qualitative conclusions concerning the concentrated-suspension regime. In fact, it should be borne in mind that the macroscopic salt gradient in the cell scale is ultimately caused by the fact that the particles carry the salt from the zone where they capture it (the co-ions onto particle surface and the counter-ions into the DDL) upon surface charging to the zone where they release it upon surface discharging. Therefore, independently of the assumption of dilute suspension, it is clear that the higher the concentration of the particles (which are effectively salt carriers), the larger the salt gradient that is generated, all other quantities (zeta potential, Debye length, particle radius, etc.) being fixed. This suggests that the anomalous behavior featured in Figures 3 and 4 at  ≳ 7 ÷ 9 should take place at significantly smaller  for concentrated suspensions.

## Conclusions

A colloidal suspension of particles that acquire surface charge by sequestration of ions (e.g., hydrogen ions) from the surrounding electrolyte solution features remarkable electrophoretic behavior. It is shown for the first time that when the surface charge acquired is moderately large, the effective mobility can be enormously larger than the ordinary Smoluchowski mobility. Even more surprisingly, in certain conditions, two critical values of zeta potential exist, between which mobility reversal occurs (i.e., the mobility becomes negative). Behind such counterintuitive behavior is a salt gradient arising in the bulk electrolyte. This gradient (which dominates the phoretic drift) is not externally applied but is engendered within the process, stemming from the charging and discharging of the particles at separate locations in the electrolyte solution, where the salt is, respectively, sequestered and liberated by the particles themselves. For critical values of parameters *Z*_,_ λ/*R*, and , the onset of this salt gradient can determine the vanishing of the electric field in the suspension, hence the divergence of the electrophoretic mobility. Remarkably, ranges of these parameters are identified wherein the electric field in the suspension is inverted with respect to the externally applied field (at the electrodes), which results in reversal of the electrophoretic mobility.

These results have potential impact on some current technical applications. For example, the strong applied voltages generally required in electrophoresis technologies may be an issue.^3^ Clearly, for a required electrophoretic rate, the huge mobilities (i.e., small ∇Ψ_∞_) predicted by this theory enable smaller applied fields with correspondingly improved efficiency of the process. Another aspect concerns the very high sensitivity of mobility with respect to zeta potential and particle size (via the λ/*R* parameter). This has clear implications in the electrophoretic separation of particles suspensions with polydisperse size and/or slightly different zeta potential.

Moreover, these findings should spur fundamentally new ideas and approaches to harness or control physical processes driven by chemical gradients and/or an electric field. These processes play an essential role in the functioning of a variety of natural systems.^41^ They are also central to a number of emerging technologies, especially in the micro-/nanofluidic area.^6,42−45^ We expect the present results to have an impact in such broader contexts as well.

It has been shown how, even in the absence of an externally-applied salt gradient or electric field, merely placing such kind of colloidal suspension into an electrolyte solution automatically gives rise to large-scale, self-sustaining salt gradients, which, in turn, cause a phoretic drift of the suspension itself. Apparently, this represents a new type of autophoresis. In fact, remarkably, because the salt gradient sets up on a large scale (as compared to the size of particle), the resulting autophoretic drift concerns not just individual particles but the particle suspension as a whole. It is worth exploring these new aspects and their possible applications.

Finally, the fact that the electric field in the bulk suspension can be made vanishing or even opposed to the voltage difference applied externally to the electrodes is unedited and intriguing and, as such, should spur ideas to conceive radically new applications. We hope that the new features introduced by the present theoretical study will prompt experimental validation in the near future.

## Appendix A: Averaged Ion Flux *F*

Our goal here is obtaining the expression for ***F*** (cf. eq 4), the average flux density of inert ions in the (electroneutral) fluid domain external to the DDL. The local flux density outside the DDL is

A1

where

A2a

or

A2b

depending on whether the spectator ion species is, respectively, the counter- or the co-ion (note that *c*_∞_ in place of *c* was used in eq A2 in force of the weak-field hypothesis). We denote by *V_m_* a sample volume containing *m* particles, where *m* is a large integer number (however, *V_m_* is small as compared to the electrophoretic cell). Moreover, *V_l_* (*l* = 1,2, ...*m*) denotes the space occupied by the *l*th particle present in *V_m_* together with its own DDL, while *S_l_* indicates the surface enfolding *V_l_*. Finally, we indicate by *V_U_* and *S_U_*, respectively, the union of *V_l_* and of *S_l_* (). With this notation, ***F*** is just the average of ***f*** over *V_m_* excluding space *V_U_*. Using eq A1,

A3

We have

A4

where by definition,  is just the average field in the suspension. Then, our task is to calculate the integral on the *rhs* of eq A4. It is useful to recall here some of the assumptions made earlier. First, the particles are considered as randomly distributed in the electrolyte medium so that the suspension is statistically isotropic. As a result, the average field ⟨Φ⟩ is independent of the shape and size of the sample volume *V_m_*. Further, we consider that the particles are far from one another (*Z* ≪ 1), whereby field interaction between particles can be ignored. Finally, we suppose spherical particles all of radius *R*. Under these assumptions, the field surrounding each particle is same as for the one-particle problem, i.e., single sphere (of radius *R*) in infinite space with far-distance field Φ_∞_ uniform and equal to ⟨Φ⟩.^34,35^ This is true, in particular, at the particle surface. Formally, this fact is expressed as follows:

A5

where ***x***_*l*_^c^ is position vector of the center of the *l*th particle. Suffix 0 denotes reference to the one-particle problem. We now employ the Gauss theorem in the following version:

A6

where *V* is an arbitrary space domain and *S* its surface boundary, ***n*** is the unit external normal to *S*, and ***e***_*i*_ (*i* = 1,2,3) the unit vectors along the coordinates. Applying eq A6 to space *V_U_* occupied by all *m* particles (and their DDLs) and using eq A5, we have

A7

In order to proceed, we have to use the proper expression for Φ_0_(***x***). According to eq A2, aside from an unimportant additive constant, this is

A8

where either the – or + sign applies as prescribed just after eq A2. It should be borne in mind that *c*_0_(***x***) and Ψ_0_(***x***) are the solutions for the one-particle problem (probe sphere). However, these are provided, respectively, by eqs 11 and 12. We substitute these expressions into eq A8 and, subsequently, the latter into the *rhs* of eq A7. Then, we perform surface integration over sphere *S*_0_. The result is

A9

where *V*_0_ () is particle volume, while (1 + *K*)Ψ_∞_ and (1 + *Q*)*c*_∞_ are expressed, respectively, by eqs 13 and 14 (as proper linear combinations of Ψ_∞_ and *c*_∞_). We now substitute eq A9 into eq A4 and, subsequently, the latter into eq A3. Then, after noting that *mV*_0_/*V_m_* is just the volume fraction of the particles *Z*, we arrive at the sought expression for the average flux density of inert ions in the (electroneutral) fluid domain external to the DDL:

A10a

A10b

depending on whether the spectator-ion species is, respectively, the counter- or the co-ion. Finally, substituting eqs 13 and 14, respectively, for (1 + *K*)Ψ_∞_ and (1 + *Q*)*c*_∞_ in eq A10, we arrive right at eq 22.

## Appendix B: Collinearity of the ∇c_∞_ and ∇Ψ_∞_ Fields

In the resolution of the differential system eqs 6–10, we assumed the vectors *c*_∞_ and Ψ_∞_ to be collinear. This assumption is certainly verified for the unidimensional cell configuration considered in Figure 1a. However, as shown here, the above assumption is valid in general. More specifically, we show how condition 4 forces the two vectors to be always collinear irrespective of the particular cell geometry.

Given the linear and homogeneous structure of system eqs 6–10, its general solution is obtained by superposing the solution for far field conditions: Ψ → Ψ_∞_, *c* → **0** as *x* → ∞ with the solution for far field conditions: Ψ → **0**, *c* → *c*_∞_ as *x* → ∞. As a consequence, given that the surface slip velocity ***u***_*S*_ is expressed by eq 5 as linear combination of _*S*_Ψ and _S_*c*, the particle velocity ***U*** must also be expressed a linear combination of Ψ_∞_ and *c*_∞_:

B1

with *a* and *b* proper dimensional scalar constants. Incidentally, these constants can be determined by direct resolution of system eqs 6–10 and subsequent substitution into eq 5; however, that is not necessary to our scope here. We now turn to eq 4. First, we note, in view of eqs A3 and A2, that ***F*** must also result as a linear combination of Ψ_∞_ and *c*_∞_. Then, substituting such combination into eq 4 and recalling eq 21, we have:

B2

with *â* and *b̂* proper dimensional scalar constants, which can be determined, but again, that is not necessary here. Instead, it is important to observe that eqs B2 and B3 are independent from each other as the first is just the solution of the problem of the phoretic drift of a single particle under concomitant actions of Ψ_∞_ and *c*_∞_, whereas the second one is the result of the average mass balance (eq 4) of spectator ions in the electroneutral domain of an isotropic suspension of particles migrating with velocity equal to ***U***. In fact, eq B3 involves quantities, such as the volume fraction of particle *Z*, which are not present in eq B2. Therefore, it must be *a* ≠ *â* and *b* ≠ *b̂* (incidentally, eq B2, in conjunction with eq B3, affords determination of the unknowns ***U*** and *c*_∞_ once the external field Ψ_∞_ is assigned). Coming to the point, subtracting eq B3 from eq B2, we have

B3

which shows that the two vectors Ψ_∞_ and *c*_∞_ are collinear. At this point, a glance at eqs B2 or B3 makes aware that ***U*** is also collinear with Ψ_∞_.
